# Characterization and Comparison of Ocular Surface Microbiome in Newborns

**DOI:** 10.3390/microorganisms10071390

**Published:** 2022-07-10

**Authors:** Francesco Petrillo, Arianna Petrillo, Maddalena Marrapodi, Carlo Capristo, Maria Francesca Gicchino, Paolo Montaldo, Elisabetta Caredda, Michele Reibaldi, Lara M. V. Boatti, Federica Dell’Annunziata, Veronica Folliero, Marilena Galdiero

**Affiliations:** 1 Department of Surgical Sciences, Eye Clinic Section, University of Turin, 10124 Turin, Italy; francescopetrillo09@gmail.com (F.P.); mreibaldi@libero.it (M.R.); 2 Pediatric Unit, Fondazione IRCCS “Ca’ Granda-Ospedale Maggiore-Policlinico”, 20122 Milan, Italy; ariannapetrillo30@gmail.com; 3 Department of Woman, Child and General and Specialist Surgery, University of Campania “Luigi Vanvitelli”, 80138 Napoli, Italy; maddalena.marrapodi@unicampania.it (M.M.); mariafrancesca.gicchino@unicampania.it (M.F.G.); 4 Department of Neonatology, University of Campania “Luigi Vanvitelli”, 80138 Naples, Italy; carlo.capristo@unicampania.it (C.C.); paolo.montaldo@unicampania.it (P.M.); elisabetta.caredda@unicampania.it (E.C.); 5 Arrow Diagnostics, 16152 Genoa, Italy; laramv.boatti@gmail.com; 6 Department of Experimental Medicine, University of Campania “Luigi Vanvitelli”, 80138 Naples, Italy; federica.dellannunziata@unicampania.it (F.D.); veronica.folliero@unicampania.it (V.F.)

**Keywords:** ocular surface microbiota, 16S rRNA sequencing, newborn, bacteria

## Abstract

The ocular microbiome is of fundamental importance for immune eye homeostasis, and its alteration would lead to an impairment of ocular functionality. Little evidence is reported on the composition of the ocular microbiota of term infants and on the impact of antibiotic prophylaxis. Methods: A total of 20 conjunctival swabs were collected from newborns at birth and after antibiotic treatment. Samples were subjected to 16S rRNA sequencing via system MiSeq Illumina. The data were processed with the MicrobAT software and statistical analysis were performed using two-way ANOVA. Results: Antibiotic prophylaxis with gentamicin altered the composition of the microbiota. In detail, a 1.5- and 2.01-fold reduction was recorded for *Cutibacterium acnes* (*C. acnes*) and *Massilia timonae* (*M. timonae*), respectively, whereas an increase in *Staphylococcus* spp. of 6.5 times occurred after antibiotic exposure. Conclusions: Antibiotic prophylaxis altered the ocular microbiota whose understanding could avoid adverse effects on eye health.

## 1. Introduction

The human body is an ecosystem in which bacteria play an important role in maintaining the organism’s health [1]. The microbiota can be defined as a community of microbes, including bacteria, fungi and viruses that live on or inside the organism [2]. Several extrinsic and intrinsic factors are known to modulate the human microbiota. Intrinsic factors such as pH, oxygen, nutrients and microbial interactions and extrinsic ones, including external environmental factors, such as lifestyle, aging, nutrition, surgery, exposure to antibiotics, influence the remodeling of the microbiota, inhibiting and/or selecting the growth of preferential species [3,4].

Despite several evidence reported the composition of intestinal, oral and vaginal microbiota with pathological factors and correlations, little is understood about the ocular surface microbiota (OSM).

Compared to the intestinal microbiota, with a ratio of 10 bacterial cells per eukaryotic cell, the OSM is pauci-bacterial [5]. The ocular surface contains approximately 0.06 bacteria per human conjunctival cell. This different OSM composition could be associated with the antimicrobial agents present in the tears, such as lysozyme, cationic peptides and surfactant proteins [6,7].

The first OSM investigations were based on culture-dependent methods, detecting only 50% of the species really present [8]. Due to the demanding growth conditions of some microbial species, new technologies for the OSM evaluation included culture-independent methods, via sequencing of bacterial 16S ribosomal ribonucleic acid (rRNA). The latter allows the description of the entire microbial communities with their relative abundances [9]. Through sequencing data, it emerged that *Staphylococcus* spp. are the most representative of the OSM in human adults, followed by *Propionibacterium* spp., *Corynebacterium* spp., *Streptococcus* spp. and *Haemophilus* spp. [10].

Several studies addressed the importance of healthy OSM to avoid eye diseases [11,12]. In this regard, Kim et al. reported that the Sjögren’s syndrome reduces the diversity of the ocular microbial community, affecting the abundance of bacterial strains at the phylum level [13]. Likewise, Li and Coworkers showed that the ocular surface microbiome in Chinese subjects with the dry eye was statistically different compared with Chinese subjects without a dry eye [14]. This denotes the importance of tear moisture in maintaining healthy OSM. The ocular surface microbiota in the aqueous tear deficient patients consisted of a deficient microbiota compared to the control population. A rate of 75% of aqueous tear deficient patients possessed a minimal ocular surface microbiota, comprising of 5 genera, such as *Enhydrobacter*, *Brevibacterium*, *Staphylococcus*, *Streptococcus* and *Cutibacterium* [15]. Dong and Coworkers compared the microbiome composition of the ocular surface in Chinese subjects with or without meibomian gland dysfunction (MGD). They found a significantly higher abundance of *Firmicutes* (31.7% vs. 19.7%) and *Proteobacteria* (27.5% vs. 14.7%), and a lower abundance of *Actinobacteria* (34.2% vs. 57%) in MGD vs. control patients. Patients with allergic conjunctivitis compared with an age and sex-matched control group showed a significant difference [16]. Moreover, many authors have suggested that alterations of the normal ocular microbial flora are related to several disease states, such as blepharitis, conjunctivitis, keratitis, trachoma and ocular neoplasms [17].

To date, the only studies involving the ocular surface microbiota of newborns are based on culture techniques. Initial evidence obtained through conjunctival cultures found that the lactobacillus species represented more than half of all bacteria isolated from the conjunctiva of newborns 15 min after delivery. Subsequent studies found that the composition of the microbiota changed with the mode of delivery and environmental exposure [18]. Indeed, Lee et al. noted that conjunctival cultures changed after two days of calving, regardless of calving type [19]. Many studies showed that the conjunctival microbiome identified *Firmicutes*, *Proteobacteria* and *Actinobacteria* as the main colonizers of the conjunctiva [20,21]. These findings derive from studies that analyze only the adult or mixed population. In a study by Cavuoto et al., the ocular surface microbiome of children (under 18 years of age) is dominated by *Proteobacteria* in children. They also found that *Firmicutes* and *Bacteroidetes* were the next most abundant in children. Studies conducted on an adult population found *Actinobacteria* and *Firmicutes* to be the next two most abundant phyla, after *Proteobacteria* [22]. These data suggest an age-related evolution of the ocular surface microbiome.

An in-depth study of the OSM of newborns, using recent sequencing techniques, is fundamental not only for the importance of the ocular microbiota in regulating eye homeostasis, but also for evaluating the impact of ocular prophylaxis in newborns to prevent neonatal ophthalmia, a relatively common illness, defined as conjunctivitis occurring within the first four weeks of life [23]. The most common bacteria responsible for neonatal ophtalmia are *Neisseria gonorrhoeae* and *Chlamydia trachomatis*. In Italy, prophylaxis is carried out by law (DM 11 October 1940, art.15; OJ 23 October, n.249) within one hour of giving birth and involves the use of an antiseptic solution of 1% silver nitrate or an antibacterial eye drop containing gentamicin, erythromycin or tetracyclines [24]. Of note, universal ocular prophylaxis was abandoned decades ago in several high-income countries including Denmark, Norway, Sweden and the United Kingdom. One study from the United Kingdom showed that this change did not increase the rate of blindness due to gonococcal ophthalmia [25].

Contextually, the purpose of our study was to investigate the OSM composition in newborns immediately after birth, through the sequencing of the 16S rRNA. The second phase evaluated the characteristics of OSM in a cohort of newborns after antibiotic prophylaxis, highlighting possible changes in the distribution percentages of the various phyla, genera and species.

## 2. Material and Methods

### 2.1. Study Design, Setting, Population

A prospective observational study of full-term newborns by spontaneous birth at the University Hospital of Campania “Luigi Vanvitelli” was conducted. All samples were collected from newborns at two times: (i) at birth (13 samples before antibiotic treatment); and (ii) 6 samples after antibiotic treatment. The latter was carried out following the recommendations of the committee “WHO” (World Health Organization, Genéva, Switzerland), which provided for the administration of Gentamicin (Ribomicin 0.3%, Junia Pharma, Pisa, Italy) topically, with a dosage of one drop (0.5 mL) for the eye at the time of birth. Individuals diagnosed with ocular pathologies and/or an altered general clinical picture were excluded from the two cohorts. In addition, the selection criteria included newborns whose mothers did not present urogenital tract infections. Written informed consent was obtained from parents.

### 2.2. Sample Collection

The samples were collected with a sterile applicator, without the use of an anesthetic, taking care not to touch other anatomical sites that could contaminate the swabs. The collected samples were immediately transported to a section of the microbiology laboratory, stored in a freezer at −80 °C and sequenced simultaneously at the end of the collection. A number of 19 conjunctival samples were collected in a period from November 2020 to February 2021. The methodology used for the analysis of clinical samples is the NGS sequencing method of 16S rRNA.

### 2.3. 16S Sequencing

Samples were aseptically transferred into a sterile DNase/RNase-free 2.0 mL tube for the extraction of the total DNA using the Qiamp PowerSoil kit (QIAamp^®^ DNA, Qiagen^®^, Hilden, Germany), in accordance with the manufacturer’s specifications. All samples were quantified using the Qubit fluorometer with the dsDNA high sensitivity (HS) assay kit (Invitrogen Q32854, Waltham, MA, USA) to evaluate DNA yield and normalized to 1 ng/µL. The DNA was subjected to 16S rRNA sequencing. The V1-V2-V3 regions of the 16S rRNA gene were amplified, sequenced and analyzed using Microbiota Solution A kit (Arrow Diagnostics s.r.l., AD-001.024, Genoa, Italy) and MicrobAT software (SmartSeq s.r.l., Novara, Italy). Amplification was performed in 20-µL reactions with amp mix for “PCR target” and 5 µL of DNA extract. The reactions were performed using a Veriti Thermal Cycler (Applied Biosystems^®^, #A24812, Waltham, MA, USA) following the IFU of the kit. PCR products were examined by 1.6% agarose gel electrophoresis and then purified using AMPure Beads XP (cat. n. A63880, Beckman Coulter, Inc., Brea, CA, USA). A barcode sequence was added to the ends of both the forward and reverse primers in the PCR “index”. PCR “index” products were examined and purified as described above. Amplicon targets were normalized to 10 nM and used to prepare the library for sequencing. The library was processed using kit MiSeq Reagent Nano Kit v2 (500-cycles) (cat. n: MS-103-1003, Illumina Inc., San Diego, CA, USA), following the user guidelines for the system MiSeq Illumina (“Denature ana Dilute Libraries Guide”-MiSeq System). The library was corrected by adding 10% of Phix Control (cat. n. FC-110-3001, Illumina Inc.) and sequencing using the Illumina MiSeq sequencing system (Illumina, San Diego, CA, USA). The total number of microbial assigned sequencing reads are represented in supplementary Figure S1. The data obtained were processed with the MicrobAT (Microbiota Analysis Tool) system of the SmartSeq S.r.l. (Novara, Italy) [26] using the RDP database for the taxonomy assignment (http://rdp.cme.msu.edu, accessed on 14 August 2020. Next, the sequence data was analyzed to determine alpha diversity and beta diversity using Microbiome Analyst pipeline (https://www.microbiomeanalyst.ca, accessed on 21 May 2019), in order to compare microbial population before and after antibiotic treatments. Alpha-diversity measure using Shannon index at Species level is represented as boxplot. Each boxplot represents the diversity distribution of a group. Two-D PCoA plot of Beta diversity using Bray–Curtis distance. The statistical significance of the clustering pattern in ordination plots is evaluated using Permutational ANOVA (PERMANOVA). Each axis reflects the percent of the variation between the samples, with the *X*-axis representing the highest dimension of variation and the *Y*-axis representing the second highest dimension of variation. The explained variances are shown in brackets.

### 2.4. Statistical and Bioinformatics Analysis

Statistical analysis was performed using Two-way ANOVA and graphs were generated using GraphPad Prism ver. 8.2.1 for macOS (GraphPad Software, San Diego, CA, USA, www.graphpad.com, accessed on 24 April 2019). All tests were expressed as ± Standard Deviation (SD) of 40 ocular samples.

### 2.5. Ethics Committee

Ethical approval n.416 dated 05 June 2018 was granted by the University of Campania Luigi Vanvitelli.

## 3. Results

The 16S rRNA sequencing was performed on 19 conjunctival samples from 13 full-term newborns by spontaneous birth. The patients had a mean age of 33 ± 5 years, Caucasian origin and did not present urogenital tract infections. The mean gestational time was 42 ± 2 weeks. The children were 48% and 52% male and female, respectively. The newborns underwent conjunctival swabs at birth and 7 days after antibiotic prophylaxis (Supplementary Table S1).

### 3.1. Phylum

**Figure 1:**
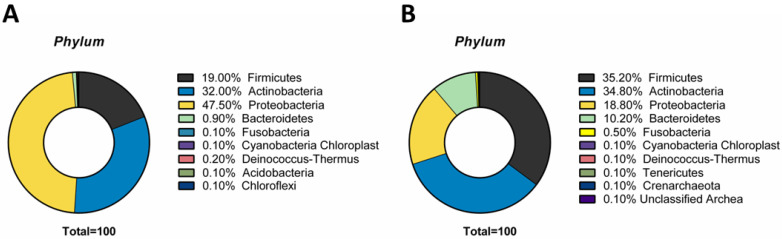
(**A**) Phylum of conjunctival samples performed at birth and (**B**) after antibiotic prophylaxis (*p*-value < 0.0001).

At the phylum level, the composition of the conjunctival microbiome at birth was dominated by *Proteobacteria* (47.5 ± 0.8%), followed by *Actinobacteria* (32.00 ± 1.2%) and *Firmicutes* (19 ± 0.6%). In minority percentage, *Bacteroidetes* (0.9 ± 0.2%) and *Deinococcus* (0.2 ± 0.01%) were recorded (Figure 1A). After topical treatment with gentamicin, significant qualitative and quantitative changes were verified. In detail, *Firmicutes* (35.2 ± 2.0%) become the predominant phylum, followed by *Actinobacteria* (34.80 ± 1.1%) and *Proteobacteria* (18.80 ± 1.2%). A slight increase in *Bacteroidetes* (10.2 ± 0.8%) and *Fusobacteria* (0.5 ± 0.04%) occurred (Figure 1B).

### 3.2. Genus

**Figure 2:**
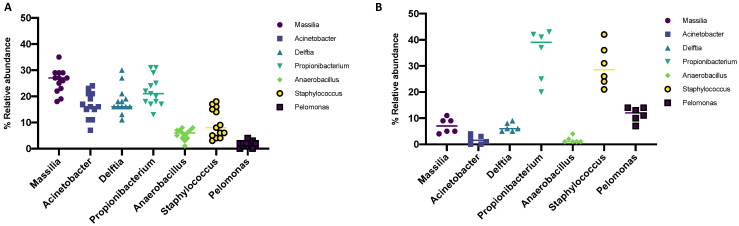
Distribution of relative genera abundance in conjunctival swabs before (**A**) and after (**B**) prophylaxis with gentamicin (*p*-value < 0.0001).

Antibiotic treatment caused a significant 3.2, 6.4, and 2.4-fold reduction of the genera Massilia, Acinetobacter and Delftia, respectively, compared to the control population (Figure 2A,B). The relative abundance of *Propionibacterium* and *Anaerobacillus* did not record significant changes while a total depletion of the genus Pelomonas was verified. On the other hand, the *Staphylococcus* genus showed an increase of 3 times after antibiotic prophylaxis, becoming the predominant genus in the OSM. Furthermore, the appearance of the *Streptococcus* genus, not present at birth, was recorded.

### 3.3. Species

**Figure 3:**
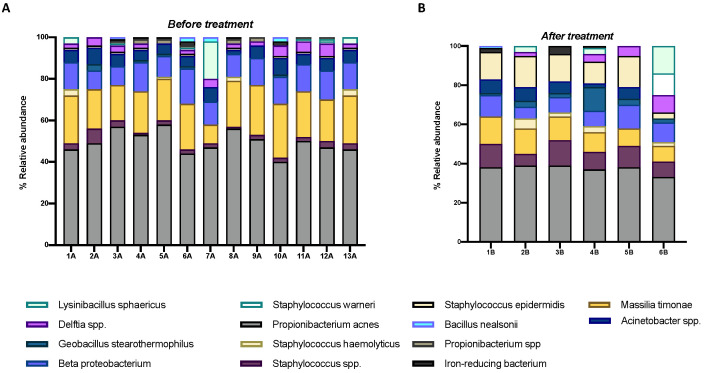
Distribution of relative species abundance in conjunctival swabs before (**A**) and after (**B**) prophylaxis with gentamicin (*p*-value < 0.0001).

The 16S rRNA sequencing data allowed the identification of the main species in newborns (Figure 3). *Cutibacterium acnes* (*C. acnes*) was the most representative species, with an average relative abundance of 52%. This strain is a normal resident of human skin and constitutes the predominant OSM species in healthy subjects [27]. After prophylactic treatment, the average relative abundance of the above species was 36.5 ± 2.4%. Therefore, a noticeable reduction of commensal *C. acnes* was observed, with a 1.5-fold decrease, compared to the untreated population. The second predominant species in ocular samples was *Massilia timonae* (*M. timonae*) (21.4 ± 4.0%), a common OSM commensal of healthy individuals [27]. This species undergoes a significant reduction after antibiotic prophylaxis, registering a decrease rate of 2.11. Moreover, an increase in *Staphylococcus* spp., in particular *Staphylococcus epidermidis* (*S. epidermidis*), was verified with an average relative abundance of 4.1 ± 1.3 and 26.2 ± 3.5%, before and after treatment with gentamicin, respectively. No significant changes were found for *Acinetobacter* spp. and Beta-proteobacteria.

### 3.4. Evaluation of Microbial Diversity

**Figure 4:**
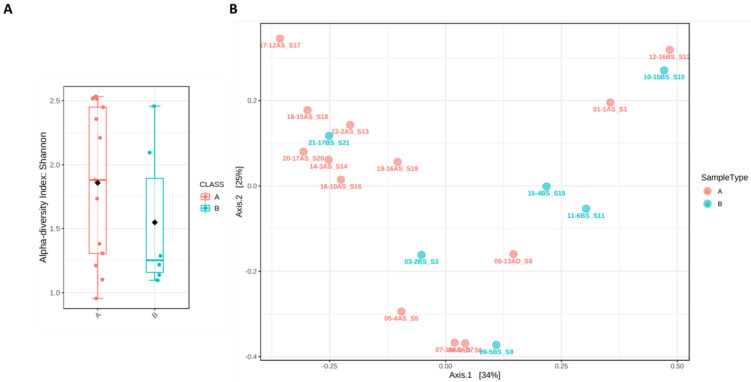
Determinations of alpha (**A**) and beta diversity PCoA (**B**). Alpha-diversity measure using Shannon index at Species level is represented as boxplot; each boxplot represents the diversity distribution of a group (Shannon index values are reported in Figure S2). Beta diversity is represented as a 2-D PCoA plot using Bray–Curtis distance. The statistical significance of the clustering pattern in ordination plots is evaluated using Permutational ANOVA (PERMANOVA). Each axis reflects the percent of the variation between the samples with the *X*-axis representing the highest dimension of variation and the *Y*-axis representing the second highest dimension of variation. The explained variances are shown in brackets.

Reduced microbiota diversity associated with antibiotic treatment has been abundantly demonstrated in humans. This result was proven in our study by comparing the composition of the microbiota of newborn babies before and after prophylactic treatment. Compared to the newborns without prophylactic treatment, the ocular microbiota diversity and richness of the newborns after antibiotic treatment was reduced between samples before and after antibiotic treatment. Data was analyzed to determine alpha diversity and beta diversity. The first is used to measure the diversity present within a sample or community. This method can be characterized via the total number of genera (richness), the abundances of the genera (evenness) or measures that considered both richness and evenness. How these measures estimates the diversity is need to be considered when performing alpha diversity analysis. Beta diversity provides a way to compare the diversity or composition between two samples or microbial communities. These methods compare the changes in the presence/absence or abundance of thousands of genera present in a dataset and summarize these into how ’similar’ or ’dissimilar’ two samples. Each sample gets compared to every other sample generating a distance or dissimilarity matrix. Both parameter showed no statistically significant changes (alpha diversity, *p*-value of 0.28181; beta diversity, *p*-value less than 0.266), underlining that, at the microbial population level, there are no major differences before and after antibiotic treatment in the child (Figure 4).

## 4. Discussion

The human microbiome plays an important role in human health and influences the development of diseases [1]. Understanding the composition of the microbiota would have an impact on the management and treatment of various disorders [28]. In most studies, constituents of the microbiota are identified through next-generation sequencing platforms applied to amplicons of the 16S ribosomal RNA (rRNA) gene [29]. This has revolutionized the study of microbiota diversity in different districts and conditions. However, short 16S rRNA sequences often restricted the taxonomic classification of the microbial community to the genus and species level [30]. Here, we investigated the ocular surface microbiome in full-term newborns by spontaneous birth.

The adults and children OSM analysis has been a subject of study for several years [31,32]. The microbiota variation over time is a key factor in understanding possible connections between dysbiosis and the onset of related ocular surface diseases [5]. However, the correct temporal evolution analysis of the microbiota needs the knowledge of the OSM composition at birth and to date, no study described the OSM in newborns.

Our evidence showed, in agreement with other studies, that OSM was paucibacterial, but with a predominance of *Proteobacteria*, *Actinobacteria* and *Firmicutes* phyla [33,34]. Figure 1 shows a difference in the percentage of the *Bacteroidetes* group. Since this bacterium is anaerobic, it is quite strange to our knowledge that such a percentage was found in such material and therefore needs clarification. Otherwise, in a previous study conducted considering a cohort with age <18 years, the analysis of 16s rRNA revealed that the majority of the organisms were *Proteobacteria*, *Firmicutes* and *Bacteroidetes* [10]. This difference underlined that the OSM at birth underwent variations in the abundance of phyla. The explanation for this variability could be threefold: (i) the environmental variations to which a child is subjected are multiple compared to the newborn [35,36]; (ii) the diet and hand-to-eye contact of infants is different with respect to the child [37]; (iii) during the first years of life, children are frequently subject to surgery or exposure to antibiotics [38,39]. All these factors taken together can contribute to determining changes in the OSM. Furthermore, the authors subjected the V4–V5 region of bacterial 16S rRNA to characterize the microbiota, while the V1–V3 regions were sequenced in the present study. This could explain some of the differences in the OSM composition [10]. Another study conducted by Zhou et al., included 105 conjunctiva samples from children (*n* = 21) and adults (*n* = 84) and analyzed the V1–V3 region of 16S rRNA. The result reported that OSM was composed of *Actinobacteria* (46%), *Proteobacteria* (24%) and *Firmicutes* (22%). However, the composition of the pediatric microbiota was not described separately from the adult one [40]. Eder et al. have evaluated the spectrum of normal conjunctival flora in newborns with culture technique. Among the vaginally delivered newborns, the most common bacteria isolated were coagulase-negative Staphylococcus (38%), *Propionibacterium* spp. (20%) and *Corynebacterium* spp. (16%) [41]. Our study confirms the presence of *Staphylococcus* and *Propionibacterium* spp. as predominant species, but, thanks to the use of recent gene sequencing techniques that allow us to overcome the limitations of culture techniques, highlights the existence of a much wider commensal flora.

Respect with to newborn OSM, the microbiota of adults was extensively investigated [27,42]. Several evidences report the Proteobacteria as the main phylum in the ocular microbiota of adults. Our data prove that *Actinobacteria* and *Firmicutes* are the two most abundant phyla in the ocular microbiota of newborns. This suggests that the neonatal ocular microbiota could undergo changes over time [43,44]. Nevertheless, in order to support this hypothesis, further studies are necessary by monitoring, at different ages, the OSM evolution of the same cohort. 

In the second phase of the study, we evaluated the patients tested after antibiotic prophylaxis with gentamicin. On the seventh day after treatment, infants underwent right and left conjunctival swabs. Data of 16S rRNA sequencing revealed significant changes in the OSM composition, with a predominance of *Firmicutes*, *Actinobacteria* and *Proteobacteria* phyla. Therefore, the majority shifted in favor of the *Firmicutes*, which represented the minority of the phylum, before treatment. On the other hand, the *Proteobacteria* underwent a decrease of over 50% after antibiotic prophylaxis, becoming the less abundant Phylum. About genera analysis, Ozkan et al. reported that *Corynebacterium*, *Acinetobacteria, Pseudomonas*, *Delftia*, *Streptococcus*, *Massilia* and *Rothia* represented 80% of the microbial genera most represented on the ocular surface in adults [21]. Our data showed that at birth, the genera *Massilia*, *Acinetobacter* and *Delftia* predominate over the conjunctiva. The latter were drastically reduced by prophylactic treatment. Conversely, exposure to antibiotics caused an increase in the *Staphylococcus* genus. Consistent with our findings, Dave et al. reported a substantial change in ocular flora after antibiotic treatment. Detailed in untreated patients *S. epidermidis* and *S. aureus* accounted for 54.5% and 18.2% of OSM, respectively. The treatment with azithromycin involved an increase of *S. epidermidis* and *S. aureus* constituting 90.9% and 4.5% of the microbiota, respectively. Studies have shown the impact of antibiotics on bacterial biofilm formation. Wang et al. reported a significant increase in biofilm formation of *S. epidermidis* strains after antibiotic treatment [45]. The presence of the polysaccharide matrix of the biofilm limits the spread of the antibiotic, giving resistance to the treatment and the possibility of bacterial replication. Moreover, treatment with fluoroquinolones reduced the rate of Gram-negative species from 8.7% to 1.6% [46]. This evidence demonstrated that, although ophthalmic antibiotics are used to treat and prevent a variety of infectious conditions, their disproportionate use could negatively alter the eye surface microbiota, contributing to opportunistic pathogenic species invasion and eye diseases.

Like all studies, our findings should be considered together with several limitations. Firstly, 16S rRNA sequencing is subject to background noise, due to possible sampling errors and contamination. Secondly, the cohort being analyzed was small, since it was a study focused on the neonatal OSM of the “Luigi Vanvitelli” hospital. For this reason, patients can be considered representative of the surrounding community, but not of the general population. Subsequent investigations will be carried out to translate this study into different geographical sites, recruiting samples from different Italian regions to establish a national pilot study. Finally, the choice of full-term newborns by spontaneous birth does not allow us to extend the results to those born with cesarean section and/or in different gestational periods. For this reason, after analyzing on a national scale the OSM of newborns with natural births, our study will focus on the changes that are established in the case of cesarean and/or premature birth.

Despite these limitations, our data add a piece to the growing understanding of the ocular microbiome, evaluating for the first time OSM directly to birth, which has never been investigated to date. Furthermore, the study highlights variations that occurred after normal hospital prophylactic practice, which could affect ocular health.

## 5. Conclusions

The ocular surface microbiota plays an essential role in maintaining homeostasis and immunity of the eye. Several factors contribute to altering the ocular microbiota, changing its composition. Alteration of the microbiota composition exposes the eye to colonization by pathogens. The microbiome of the healthy conjunctiva is made up of bacteria commonly classified as opportunistic pathogens. Some of these bacteria can take over, overcome the host’s innate immunity barrier through quorum sensing phenomena, generating disease. A case in point is represented by *S. aureus*. Understanding the composition of the healthy ocular microbiota and how it changes with antibiotic treatment could be crucial in avoiding the development of infectious and non-infectious diseases.

## Data Availability

The data presented in this study are available on request from the corresponding author.

## Supplementary Materials

The following supporting information can be downloaded at: https://www.mdpi.com/article/10.3390/microorganisms10071390/s1, Table S1: Characteristics of the cohort subjected to study; Figure S1: Read counts for each compound; Table S2: Percentage values of assigned and unassigned reads at genus level.
