# Supplementary material for: Characterization and Comparison of Ocular Surface Microbiome in Newborns
Source: Microorganisms. 2022 Jul 10;10(7):1390. doi: 10.3390/microorganisms10071390 (PMC9320102; doi:10.3390/microorganisms10071390)
Supplement: Supplementary file 1 [file microorganisms-10-01390-s001.zip › microorganisms-1777616-supplementary.pdf]

microorganisms

MDPI

Supplementary Material

Supplementary Table S1. Characteristics of the cohort subjected to study.

ID sample	Sex	Gestational age	Ethnicity	Vaginal swab	Ocular swab (dx and sx)
**1A**	F	40 weeks	Caucasian	negative	T0: at birth
**1B**					T1: gentamicin prophylaxis
**2A**	F	44 weeks	Caucasian	negative	T0: at birth
**2B**					T1: gentamicin prophylaxis
**3A**	F	46 weeks	Caucasian	negative	T0: at birth
**3B**					T1: gentamicin prophylaxis
**4A**	F	46 weeks	Caucasian	negative	T0: at birth
**4B**					T1: gentamicin prophylaxis
**5A**	M	42 weeks	Caucasian	negative	T0: at birth
**5B**					T1: gentamicin prophylaxis
**6A**	M	42 weeks	Caucasian	negative	T0: at birth
**6B**					T1: gentamicin prophylaxis
**7A**	F	42 weeks	Caucasian	negative	T0: at birth
/					T1: /
**8A**	F	43 weeks	Caucasian	negative	T0: at birth
/					T1: /
**9A**	F	39 weeks	Caucasian	negative	T0: at birth
/					T1: /
**10A**	M	41 weeks	Caucasian	negative	T0: at birth
/					T1: /
**11A**	M	42 weeks	Caucasian	negative	T0: at birth
/					T1: /
**12A**	M	40 weeks	Caucasian	negative	T0: at birth
/					T1: /
**13A**	M	42 weeks	Caucasian	negative	T0: at birth
/					T1: /

Supplementary Figure S1. Read counts for each compound.

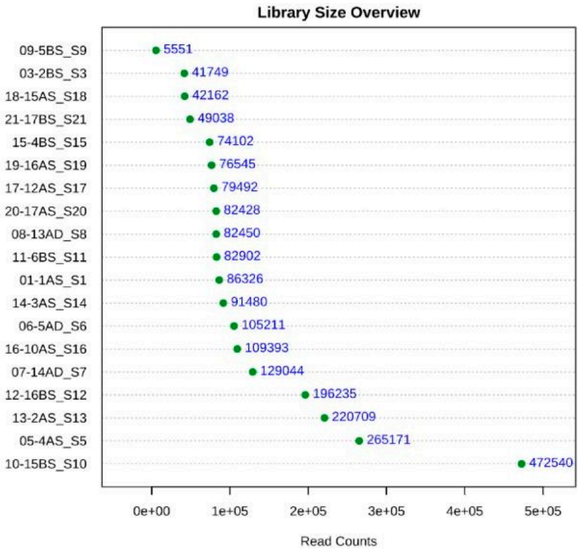

Supplementary Table S2. Percentage values of assigned and unassigned reads at genus level.

Name	Reads assigned (% value)	Reads unassigned (% value)
1-A	92.53	7.48
2-A	92.46	7.55
2-B	93.59	6.43
3-A	90.33	9.64
4-A	95.49	4.53
4-B	75.81	24.11
5-A	94.85	5.16
5-B	96.70	3.32
6-B	96.49	3.51
10-A	91.01	9.00
12-A	87.39	12.60
13-A	93.63	6.38
14-A	95.69	4.31
15-A	89.74	10.29
15-B	96.84	3.15
16-A	90.68	9.27
16-B	97.15	2.83
17-A	91.92	8.06
17-B	85.63	14.36
**Mean (%)**	92.00	8.00
**Std.Dev. (%)**	5.09	5.07
